# Disability-related barriers to dental care among older Canadian adults: the role of sex as an effect modifier

**DOI:** 10.3389/froh.2026.1835484

**Published:** 2026-06-16

**Authors:** Anil Menon, Marziyeh Shafizadeh, Abdulrahman Ghoneim

**Affiliations:** 1 Department of Preventive Dental Science, Dr. Gerald Niznick College of Dentistry, Rady Faculty of Health Sciences, University of Manitoba, Winnipeg, MB, Canada; 2 Faculty of Dentistry, Dalhousie University, Halifax, NS, Canada

**Keywords:** Canadian community health survey, Canadian dental care plan, dental care access, disability, effect modification, health equity, older adults, sex

## Abstract

**Background:**

Older adults with functional disabilities experience challenges accessing dental care, yet the role of sex as an effect modifier of disability-related access barriers remains underexamined in Canada. The implementation of the Canadian Dental Care Plan (CDCP) creates an urgent need for pre-policy baseline evidence to guide targeting and evaluation.

**Methods:**

This cross-sectional study investigates whether sex affects the relationship between functional difficulty (used here as a broad proxy for disability) and three aspects of dental care access among Canadian adults 65+: cost-related visit and treatment avoidance, and emergency-only or no dental visits. Analyzing 23,967 Canadian Community Health Survey (CCHS) 2022 participants, disability was defined as having at least some difficulty in one or more functional domains. Survey-weighted logistic regression estimated adjusted odds ratios, controlling for income, dental insurance, Indigenous identity, and immigration. Functional difficulty-by-sex interactions were tested, and predicted probabilities calculated using marginal means.

**Results:**

Functional difficulty was linked to higher odds of access barriers: dental visit avoidance due to cost (OR 1.58), dental treatment avoidance due to cost (OR 1.59), and emergency-only or no dental visits (OR 1.60), all *p* < 0.001, even after adjusting for income and insurance. No significant functional difficulty-by-sex interactions were detected, with a consistent 7–8 percentage point disability gap in cost-related avoidance for both sexes. Women had lower odds of emergency-only or no dental visits (OR 0.68, *p* < 0.001). Dental insurance was the strongest protective factor, and immigration increased cost-related visit and treatment avoidance (OR 1.58 and 1.39, respectively).

**Conclusion:**

Functional difficulty was linked to reduced access across all three outcomes, with little evidence of differences between male and female groups. These findings support inclusive accommodation strategies within the CDCP and suggest monitoring sex-related differences in emergency-only or no dental visits during implementation and evaluation.

## 1 Introduction

Good oral health matters far beyond the mouth. It affects what people can eat, how they communicate, and how they feel about themselves, yet access to dental care in Canada has long been shaped less by clinical need than by financial circumstance. Older adults are particularly exposed to this gap: most lose employer-sponsored dental coverage when they retire, and unlike visits to a physician or nurse, dental care sits almost entirely outside provincial public health insurance (1, 2). For older adults living with functional difficulties, the problem is compounded. Cost is a barrier for many Canadians, but disability adds a second layer of difficulty, navigating appointments, managing transportation, and communicating with providers, which money alone cannot always resolve.

The Canadian Dental Care Plan (CDCP), introduced in 2023 and progressively expanded through 2025, is the most ambitious federal oral health initiative Canada has ever undertaken. For uninsured Canadians with household incomes below $90,000, it offers something previously unavailable: publicly funded dental coverage (3). For older adults who retire without carrying over dental benefits, this is a significant shift. A program's coverage is only as equitable as its reach; therefore, understanding which groups are excluded and the reasons for their exclusion during the planning stage is paramount to assessing the program's overall utility.

Older adults with functional difficulties deserve special attention in this context. Functional difficulty, whether it involves mobility, cognition, hearing, vision, or self-care, is associated with reduced ability to navigate dental care systems, higher rates of oral disease, and greater reliance on others to coordinate health needs (4, 5). These challenges do not exist in isolation: they are compounded by the fact that disability is more prevalent in lower-income households and those least likely to hold private insurance (6). Those facing the greatest practical barriers to dental care are often also those with the fewest financial means to overcome them. Despite the scale of this problem, population-level evidence from Canada that directly quantifies it, especially in the window just before the CDCP came into force, remains sparse.

Sex is a recognized social determinant of oral health, shaping patterns of disease, care-seeking behaviour, and treatment experience over the life course (7, 8). Older women and men arrive at later life through different pathways; women are more likely to have had lower lifetime earnings, to live alone in older age, and to report functional difficulty, all of which could amplify the barriers that disability creates around dental care access (9–11). Whether sex modifies the relationship between disability and those access barriers, that is, whether the disability penalty is meaningfully larger or smaller for women than for men, has not been formally tested in a nationally representative Canadian sample.

This study aims to 1) estimate how strongly functional difficulty is associated with cost-related dental visit avoidance, treatment avoidance, and emergency-only or no dental visits among Canadians aged 65 and older; 2) examine whether sex modifies those associations; and 3) describe the independent contribution of sex to dental care-seeking patterns in this population. We will discuss our findings in the context of the CDCP and its potential impact on dental care for older adults with functional difficulties.

## 2 Materials and methods

### 2.1 Data source and study population

We conducted a cross-sectional analysis using data from the immediately pre-CDCP rollout, Canadian Community Health Survey (CCHS) 2022, Annual component, Public Use Microdata File (PUMF) (12). The CCHS is a nationally representative, cross-sectional survey of non-institutionalized Canadians aged 12 and over living in private dwellings across all provinces and territories. It employs a complex, multi-stage, stratified sampling design to produce reliable estimates at the health region level. In 2022, the CCHS underwent a major redesign, transitioning from computer-assisted telephone and in-person interviewing (CATI/CAPI) to a primarily online electronic questionnaire (EQ) format, with CATI/CAPI follow-up for nonresponse. Users are advised to exercise caution when making direct comparisons with pre-2022 CCHS cycles. The overall response rate for the 2022 CCHS was 42.7%.

The analytic sample was restricted to respondents aged 65 years and older. Participants with missing data on disability status, any dental care access outcome, household income, dental insurance status, or immigration status were excluded from the primary analysis. All estimates met the Statistics Canada PUMF minimum release threshold of 30 observations per cell.

### 2.2 Exposure: functional difficulty

The primary independent variable was self-reported functional difficulty (disability), derived from the Washington Group Short Set on Functioning (WDM module), which was administered as theme content in the 2022 CCHS across all ten provinces. Disability was operationalized as a binary variable indicating the presence of at least “some difficulty” in one or more functional domains (vision, hearing, mobility, cognition, self-care, and communication), vs. no difficulty in any domain. This operationalization is consistent with established practice in population-based disability research using the Washington Group instrument (13). Although functional disability measure is used as a proxy for disability in population health research, it reflects a broad and heterogeneous construct and does not correspond to clinically defined or severe disability.

### 2.3 Outcomes

Three oral health care access outcomes were examined as primary outcomes, each derived from the CCHS 2022 Oral Health modules (OHM and OHM3), which were introduced as theme content in this cycle: (1) avoided a dental visit due to cost in the past 12 months (yes/no); (2) avoided recommended dental treatment due to cost in the past 12 months (yes/no); and (3) emergency-only or no dental visits, defined as a binary indicator derived from the dental visit frequency variable (respondents who reported visiting a dental professional for emergency care only or never having visited vs. any regular or preventive pattern, including less than once a year, once a year, or more).

### 2.4 Covariates

Covariates for the primary multivariable model were selected *a priori* based on established social determinants of oral health and access-to-care frameworks, and included: household income (categorical: less than $20,000; $20,000–$39,999; $40,000–$59,999; $60,000–$79,999; $80,000 or more), dental insurance coverage (yes/no), and immigration status (immigrant/non-immigrant). Education was excluded from the primary model on the basis that household income captures substantial overlapping socioeconomic variation in this older adult sample, and to preserve model parsimony; however, education was included in the sequential sensitivity analyses (Supplementary Table S1) to confirm that its inclusion did not meaningfully alter the disability estimates. Household living arrangement (alone vs. with others) was examined descriptively given its significant sex-based distribution but was not included in the regression models because it was not identified as a primary confounder in the access-to-care framework used, and its inclusion would have introduced collinearity with income in this sample. The 2022 CCHS Public Use Microdata File includes a binary variable for sex at birth (male/female), which was used in this analysis. Sex was included as both a covariate and the primary candidate effect modifier of interest.

### 2.5 Statistical analysis

All analyses incorporated the CCHS 2022 PUMF sampling weights (WTS_M) and 500 bootstrap replicate weights to produce nationally representative, design-unbiased estimates and to account for the complex multistage stratified sampling design, as recommended by Statistics Canada. Survey-weighted logistic regression models were fitted using the survey package in R (version 4.3.2), specifying a quasibinomial family to accommodate the complex survey design. Adjusted odds ratios (ORs) with 95% confidence intervals (CIs) were estimated for each outcome. Statistical significance was defined as two-sided *p* < 0.05.

**Figure 1:**
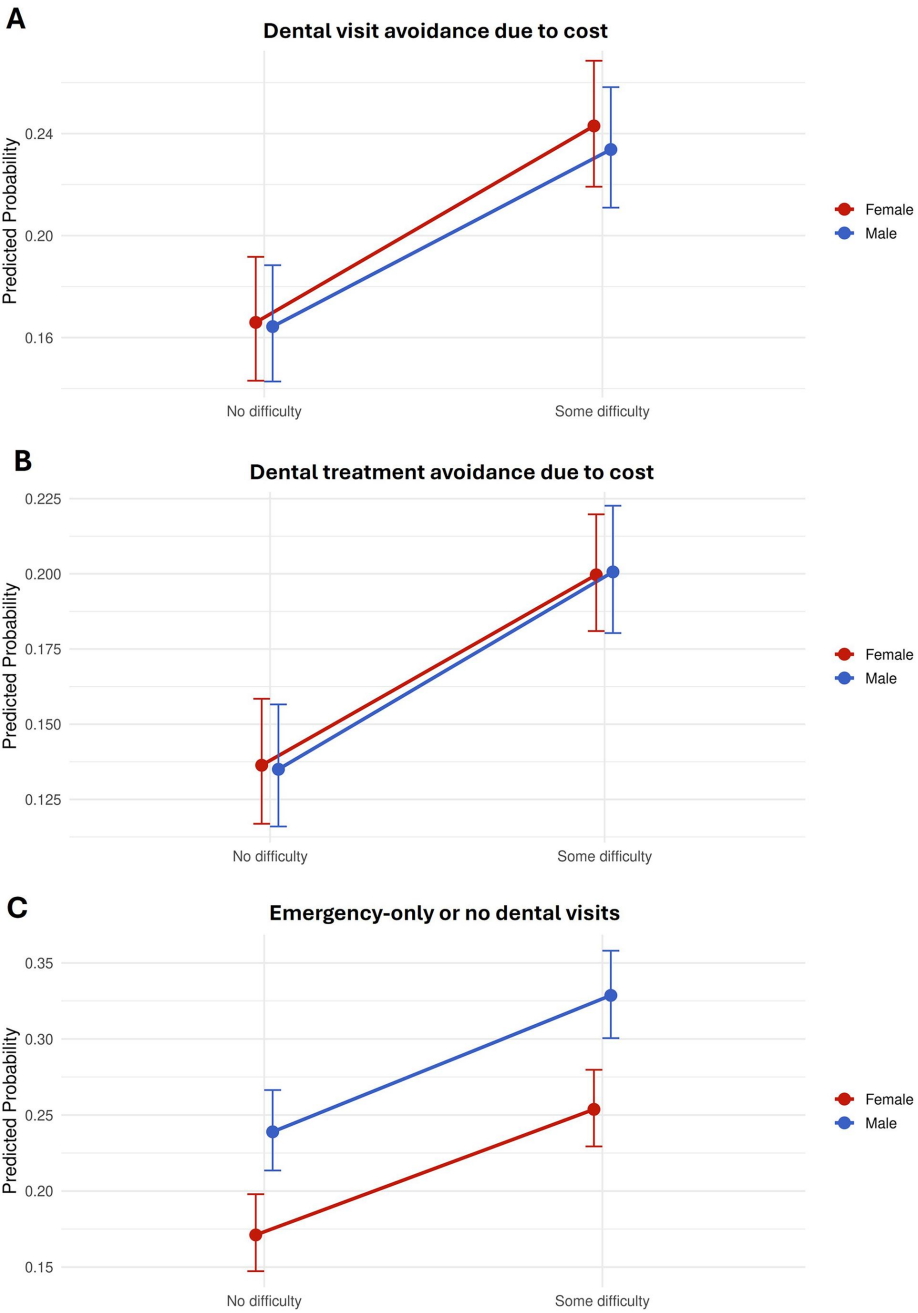
Predicted probabilities of **(A)** dental visit avoidance due to cost, **(B)** dental treatment avoidance due to cost, and **(C)** emergency-only or no dental visits by disability status and sex. Estimates are from survey-weighted logistic regression models with a Functional Difficulty × Sex interaction, adjusted for household income, dental insurance, and immigration status. Error bars indicate 95% confidence intervals.

**Figure 2:**
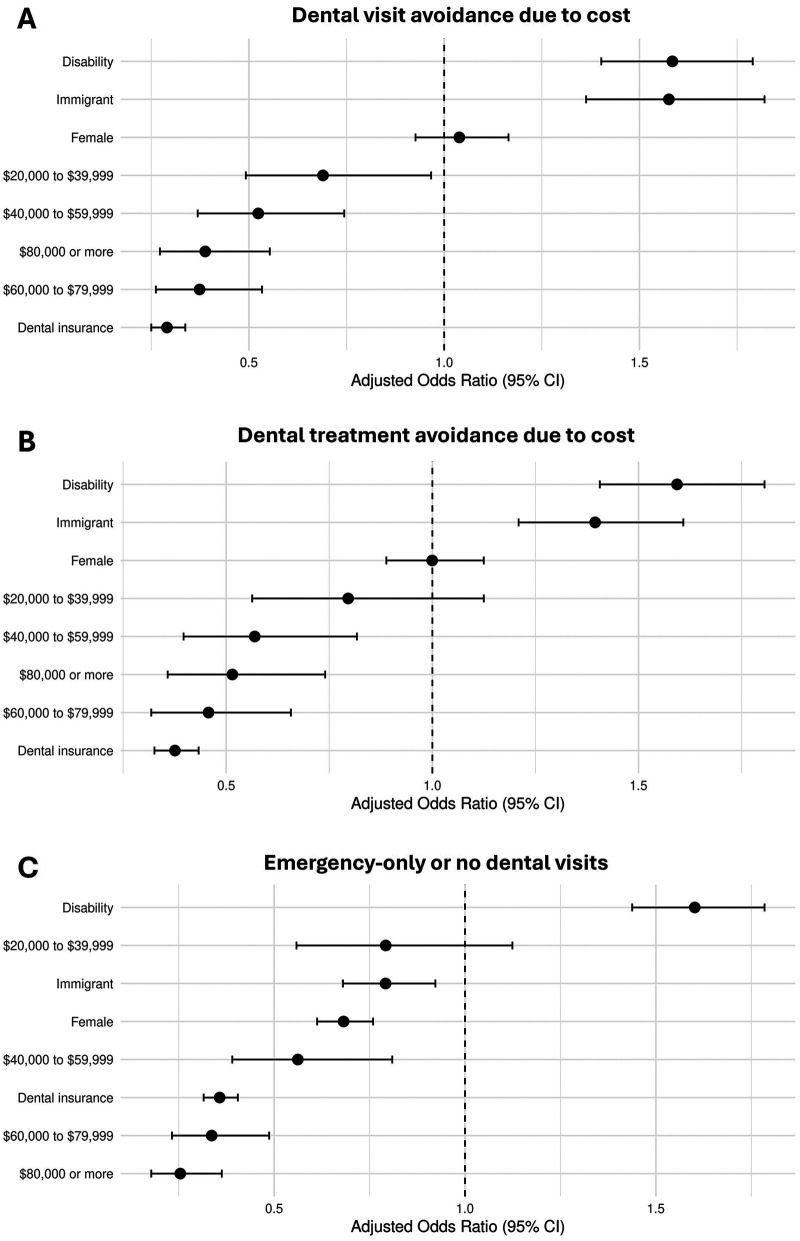
Adjusted odds ratios (ORs) and 95% confidence intervals (CIs) for associations between functional difficulty (some difficulty vs. no difficulty) for **(A)** Dental visit avoidance due to cost, **(B)** Dental treatment avoidance due to cost, and **(C)** Emergency-only or no dental visits among Canadian adults aged 65 years and older, 2022 Canadian Community Health Survey (CCHS). Models were adjusted for sex, household income, immigration status, and dental insurance.

The primary multivariable model adjusted for household income, dental insurance coverage, sex, and immigration status. Results were visualized using a forest plot, with point estimates and horizontal error bars representing 95% CIs, and a vertical reference line at OR = 1 indicating no association (Figure 1). To examine whether sex modifies the association between functional difficulty and each dental care access outcome, a Functional Difficulty × Sex interaction term was added to the fully adjusted model. Formal interaction testing was conducted for all three primary outcomes. Predicted probabilities of each outcome were estimated from the survey-weighted logistic regression models using the emmeans package in R, specifying disability status and sex as the variables of interest. Marginal predicted probabilities and their 95% CIs were derived on the response scale while holding other covariates at their observed values. Interaction plots were generated using ggplot2 to visually display the predicted probabilities across combinations of disability status and sex (Figure 2).

As sensitivity analyses, three sequential models with progressively increasing covariate adjustment were fitted for each primary outcome: Model 1, adjusted for sex only; Model 2, additionally adjusted for household income and education; and Model 3, the fully adjusted model including dental insurance. These sequential attenuation analyses are presented in Supplementary Table S1 to confirm robustness of the disability effect across model specifications.

## 3 Results

### 3.1 Sample characteristics

**Figure 3:**
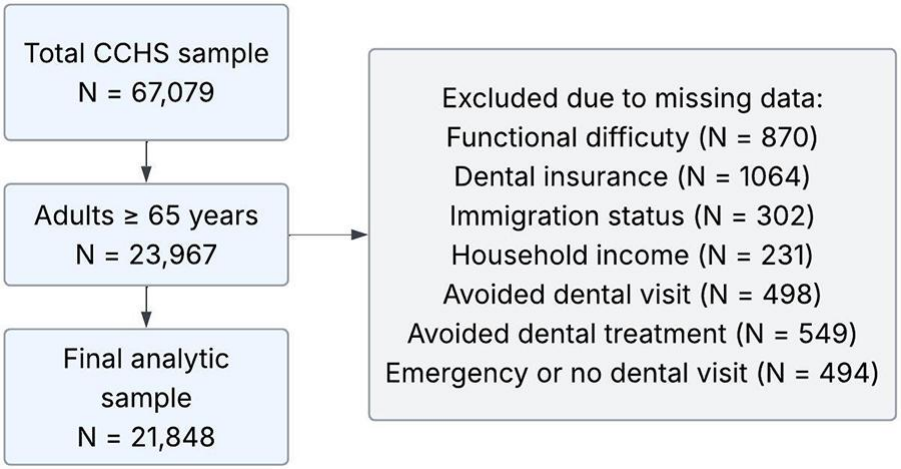
Sample flow diagram showing the inclusion of the cohort from the 2022 Canadian community health survey (CCHS).

The initial sample comprised 23,967 adults aged 65 years and older. After excluding respondents with missing data, the final analytic sample consisted of 21,848 individuals. Figure 3 presents a flow diagram of sample inclusion. The majority were female (54%), non-immigrant (82%), and non-Indigenous (98%). Nearly two-thirds (63%) reported some functional difficulty, which served as the primary exposure of interest. Forty percent of participants had dental insurance (33% private, 7.7% public). Regarding the primary outcomes, 21% reported avoiding dental visits due to cost, 18% reported avoiding recommended dental treatment due to cost, and 27% reported emergency-only or no dental-visits (Table 1).

**Table 1:** Population characteristics of adults aged 65 years and older in the CCHS. Percentages were calculated excluding missing or unknown responses.

Characteristic	*N*	*N* = 23,967^a^
Sex	23,967	
Female		13,001 (54%)
Male		10,966 (46%)
Indigenous identity	23,335	
Non-Indigenous		22,963 (98%)
Indigenous		372 (1.6%)
Province	23,967	
Alberta		2,487 (10%)
British Columbia		3,682 (15%)
Manitoba		944 (3.9%)
New Brunswick		946 (3.9%)
Newfoundland and Labrador		700 (2.9%)
Nova Scotia		1,053 (4.4%)
Ontario		8,071 (34%)
Prince Edward Island		134 (0.6%)
Quebec		4,681 (20%)
Saskatchewan		834 (3.5%)
Yukon/Northwest Territories/Nunavut		435 (1.8%)
Education	23,441	
Less than secondary school		3,448 (15%)
Secondary school		5,751 (25%)
Post-secondary school		14,242 (61%)
BMI	23,058	
Normal/Underweight		9,080 (39%)
Overweight/Obese		13,978 (61%)
Smoking	23,302	
Non-smoker		9,224 (40%)
Less than once a month		12,400 (53%)
At least once a month		27 (0.1%)
Daily		1,651 (7.1%)
Household-income	23,736	
Less than $20,000		521 (2.2%)
$20,000 to $39,999		5,467 (23%)
$40,000 to $59,999		5,093 (21%)
$60,000 to $79,999		3,671 (15%)
$80,000 or more		8,984 (38%)
Household-size	23,946	
Alone		12,536 (52%)
Two or more		11,410 (48%)
Disability	23,097	
No difficulty in any domain		8,475 (37%)
At least some difficulty in at least one domain		14,622 (63%)
Immigration-status	23,665	
Non-immigrant		19,522 (82%)
Immigrant		4,143 (18%)
Oral health	23,485	
Excellent		4,354 (19%)
Very good		8,644 (37%)
Good		7,397 (31%)
Fair		2,301 (9.8%)
Poor		789 (3.4%)
Mouth pain frequency	23,499	
Never		15,570 (66%)
Rarely		5,301 (23%)
Sometimes		2,106 (9.0%)
Often		522 (2.2%)
Dental visit frequency	23,473	
Never/Emergency only		6,394 (27%)
Less than once a year/Once a year/More than once a year		17,079 (73%)
Avoided dental visit due to cost	23,469	4,957 (21%)
Avoided dental treatment due to cost	23,418	4,196 (18%)
Avoided cleaning due to cost	23,418	2,250 (9.6%)
Avoided fillings due to cost	23,418	1,037 (4.4%)
Avoided root canal due to cost	23,418	642 (2.7%)
Avoided extraction due to cost	23,418	703 (3.0%)
Avoided tooth replacement due to cost	23,418	2,279 (9.7%)
Dental insurance	22,903	9,223 (40%)
Private dental insurance	22,903	7,589 (33%)
Public dental insurance	22,903	1,760 (7.7%)

a *n* (%).

Sex-stratified characteristics are presented in Table 2. Statistically significant sex differences were observed for household income (*p* < 0.001), with men more likely to report incomes of $80,000 or more (44% vs. 33%) and women more likely to fall in the $20,000–$39,999 range (27% vs. 18%). Women were more likely to live alone (58% vs. 46%, *p* < 0.001) and less likely to have post-secondary education (58% vs. 64%, *p* < 0.001). Women were also significantly less likely to report emergency-only or no dental visiting (25% vs. 29%, *p* < 0.001). In contrast, no significant sex difference was observed in the prevalence of functional difficulty (63% vs. 63%, *p* = 0.2), in the proportion avoiding a dental visit due to cost (21% vs. 21%, *p* = 0.2), or in the proportion avoiding recommended dental treatment due to cost (18% vs. 18%, *p* = 0.2).

**Table 2:** Sex-stratified population characteristics of adults aged 65 years and older in the CCHS. Percentages were calculated excluding missing or unknown responses.

Characteristic	*N*	Males *N* = 10,966^a^	Females *N* = 13,001^a^	*p*-value^b^
Indigenous identity	23,335			>0.9
Non-Indigenous		10,514 (98%)	12,449 (98%)	
Indigenous		171 (1.6%)	201 (1.6%)	
Province	23,967			0.3
Alberta		1,117 (10%)	1,370 (11%)	
British Columbia		1,685 (15%)	1,997 (15%)	
Manitoba		433 (3.9%)	511 (3.9%)	
New Brunswick		410 (3.7%)	536 (4.1%)	
Newfoundland and Labrador		315 (2.9%)	385 (3.0%)	
Nova Scotia		499 (4.6%)	554 (4.3%)	
Ontario		3,677 (34%)	4,394 (34%)	
Prince Edward Island		55 (0.5%)	79 (0.6%)	
Quebec		2,201 (20%)	2,480 (19%)	
Saskatchewan		363 (3.3%)	471 (3.6%)	
Yukon/Northwest Territories/Nunavut		211 (1.9%)	224 (1.7%)	
Education	23,441			**<0.001**
Less than secondary school		1,429 (13%)	2,019 (16%)	
Secondary school		2,439 (23%)	3,312 (26%)	
Post-secondary school		6,855 (64%)	7,387 (58%)	
BMI	23,058			**<0.001**
Normal/Underweight		3,710 (35%)	5,370 (43%)	
Overweight/Obese		6,991 (65%)	6,987 (57%)	
Smoking	23,302			**<0.001**
Non-smoker		3,450 (32%)	5,774 (46%)	
Less than once a month		6,374 (60%)	6,026 (48%)	
At least once a month		18 (0.2%)	9 (<0.1%)	
Daily smoker		817 (7.7%)	834 (6.6%)	
Household income	23,736			**<0.001**
Less than $20,000		225 (2.1%)	296 (2.3%)	
$20,000 to $39,999		1,972 (18%)	3,495 (27%)	
$40,000 to $59,999		2,166 (20%)	2,927 (23%)	
$60,000 to $79,999		1,739 (16%)	1,932 (15%)	
$80,000 or more		4,725 (44%)	4,259 (33%)	
Household size	23,946			**<0.001**
Alone		5,029 (46%)	7,507 (58%)	
Two or more		5,923 (54%)	5,487 (42%)	
Disability	23,097			0.2
No difficulty in any domain		3,922 (37%)	4,553 (36%)	
At least some difficulty in at least one domain		6,635 (63%)	7,987 (64%)	
Immigration status	23,665			**<0.001**
Non-immigrant		8,783 (81%)	10,739 (84%)	
Immigrant		2,040 (19%)	2,103 (16%)	
Oral health	23,485			**<0.001**
Excellent		1,857 (17%)	2,497 (20%)	
Very good		3,879 (36%)	4,765 (37%)	
Good		3,435 (32%)	3,962 (31%)	
Fair		1,163 (11%)	1,138 (8.9%)	
Poor		403 (3.8%)	386 (3.0%)	
Mouth pain frequency	23,499			**<0.001**
Never		7,094 (66%)	8,476 (66%)	
Rarely		2,571 (24%)	2,730 (21%)	
Sometimes		894 (8.3%)	1,212 (9.5%)	
Often		179 (1.7%)	343 (2.7%)	
Dental visit frequency	23,473			**<0.001**
Never/Emergency only		3,190 (29%)	3,204 (25%)	
Less than once a year/Once a year/More than once a year		7,543 (71%)	9,536 (75%)	
Avoided dental visit due to cost	23,469	2,230 (21%)	2,727 (21%)	0.2
Avoided dental treatment due to cost	23,418	1,882 (18%)	2,314 (18%)	0.2
Avoided cleaning due to cost	23,418	986 (9.2%)	1,264 (9.9%)	0.057
Avoided fillings due to cost	23,418	479 (4.5%)	558 (4.4%)	0.8
Avoided root canal due to cost	23,418	313 (2.9%)	329 (2.6%)	0.12
Avoided extraction due to cost	23,418	368 (3.4%)	335 (2.6%)	**<0.001**
Avoided tooth replacement due to cost	23,418	1,058 (9.9%)	1,221 (9.6%)	0.5
Dental insurance	22,903	4,299 (41%)	4,924 (40%)	**0.020**
Private dental insurance	22,903	3,633 (35%)	3,956 (32%)	**<0.001**
Public dental insurance	22,903	746 (7.1%)	1,014 (8.2%)	**0.004**

Bold *p*-values indicate statistical significance at α < 0.05.

a *n* (%).

b Wilcoxon rank sum test; Pearson’s Chi-squared test.

### 3.2 Functional difficulty and dental care access: main models

Results from the multivariable survey-weighted logistic regression models are presented in Table 3, with corresponding forest plots shown in Figure 1. After adjusting for household income, dental insurance, sex, and immigration status, self-reported functional difficulty was significantly associated with all three dental care access outcomes. Older adults reporting functional difficulty had 58% higher odds of avoiding a dental visit due to cost (OR 1.58, 95% CI 1.40–1.79, *p* < 0.001), 59% higher odds of avoiding recommended dental treatment (OR 1.59, 95% CI 1.41–1.80, *p* < 0.001), and 60% higher odds of emergency-only or no dental visiting (OR 1.60, 95% CI 1.44–1.78, *p* < 0.001) compared to those without functional difficulty.

**Table 3:** Association between disability and cost-related dental care avoidance and emergency-only dental visits among Canadian adults aged ≥65 years (multivariable logistic regression, 2022 CCHS).

Predictor^a^	Dental visit avoidance due to cost			Dental treatment avoidance due to cost			Emergency-only or no dental visits		
	OR	95% CI	*p*-value	OR	95% CI	*p*-value	OR	95% CI	*p*-value
Disability									
Some difficulty	1.58	1.40, 1.79	**<0**.**001**	1.59	1.41, 1.80	**<0**.**001**	1.60	1.44, 1.78	**<0**.**001**
Sex									
Female	1.04	0.93, 1.16	0.513	1.00	0.89, 1.12	0.994	0.68	0.61, 0.76	**<0**.**001**
Household income									
$20,000 to $39,999	0.69	0.49, 0.97	**0**.**031**	0.80	0.56, 1.12	0.195	0.79	0.56, 1.12	0.192
$40,000 to $59,999	0.52	0.37, 0.74	**<0**.**001**	0.57	0.40, 0.82	**0**.**002**	0.56	0.39, 0.81	**0**.**002**
$60,000 to $79,999	0.37	0.26, 0.53	**<0**.**001**	0.46	0.32, 0.66	**<0**.**001**	0.34	0.23, 0.49	**<0**.**001**
$80,000 or more	0.39	0.27, 0.55	**<0**.**001**	0.51	0.36, 0.74	**<0**.**001**	0.25	0.18, 0.36	**<0**.**001**
Dental insurance									
Insured	0.29	0.25, 0.34	**<0**.**001**	0.38	0.33, 0.43	**<0**.**001**	0.36	0.32, 0.41	**<0**.**001**
Immigration status									
Immigrant	1.58	1.36, 1.82	**<0**.**001**	1.39	1.21, 1.61	**<0**.**001**	0.79	0.68, 0.92	**0**.**003**

Bold *p*-values indicate statistical significance at α < 0.05.CI, Confidence Interval; OR, Odds Ratio.

a Reference categories: no difficulty; male sex; less than $20,000 household income; no insurance; non-immigrant.

Dental insurance was the strongest protective factor across all three outcomes, reducing the odds of cost-related dental visit avoidance by 71% (OR 0.29, 95% CI 0.25–0.34), treatment avoidance by 62% (OR 0.38, 95% CI 0.33–0.43), and emergency-only or no dental visiting by 64% (OR 0.36, 95% CI 0.32–0.41), all *p* < 0.001. A pronounced income gradient was observed for all three outcomes: compared to those with household incomes below $20,000, participants in higher income categories had substantially lower odds of cost avoidance, with ORs ranging from 0.37 to 0.69 for visit avoidance and 0.25 to 0.79 for emergency-only or no dental visiting across income strata (all higher strata *p* < 0.001 or *p* < 0.01).

Sex was not significantly associated with cost-related dental visit avoidance (OR 1.04, 95% CI 0.93–1.16, *p* = 0.513) or treatment avoidance (OR 1.00, 95% CI 0.89–1.12, *p* = 0.994). However, female sex was significantly associated with lower odds of emergency-only or no dental visiting (OR 0.68, 95% CI 0.61–0.76, *p* < 0.001). Immigration status was associated with substantially elevated odds of cost-related visit avoidance (OR 1.58, 95% CI 1.36–1.82) and treatment avoidance (OR 1.39, 95% CI 1.21–1.61), both *p* < 0.001. It was also significantly associated with emergency-only or no dental visiting (OR 0.79, 95% CI 0.68–0.92, *p* = 0.003). Sequential attenuation models confirming robustness of these associations across progressively adjusted specifications are presented in Supplementary Table S1.

### 3.3 Functional difficulty × sex interaction and predicted probabilities

Results from the interaction models are presented in Table 4. The Functional Difficulty × Sex interaction term was not statistically significant for all three outcomes: avoided dental visit due to cost (interaction OR 1.04, 95% CI 0.82–1.32, *p* = 0.752), avoided recommended dental treatment (OR 0.98, 95% CI 0.76–1.26, *p* = 0.891), and emergency-only or no dental visiting (OR 1.06, 95% CI 0.84–1.33, *p* = 0.638). The functional difficulty main effects remained significant and of comparable magnitude in the interaction models (ORs 1.55–1.61 across outcomes, all *p* < 0.001). The sex main effect for emergency-only or no dental visiting remained significant in the interaction model (OR 0.66, 95% CI 0.55–0.79, *p* < 0.001).

**Table 4:** Association between disability and cost-related dental care avoidance and emergency-only dental visits among Canadian adults aged ≥65 years, and interaction with sex (multivariable logistic regression, 2022 CCHS).

Predictor^a^	Dental visit avoidance due to cost			Dental treatment avoidance due to cost			Emergency-only or no dental visits		
	OR	95% CI	*p*-value	OR	95% CI	*p*-value	OR	95% CI	***p*-value**
Disability									
Some difficulty	1.55	1.30, 1.85	**<0**.**001**	1.61	1.34, 1.93	**<0**.**001**	1.56	1.35, 1.80	**<0**.**001**
Sex									
Female	1.01	0.83, 1.23	0.905	1.01	0.82, 1.25	0.915	0.66	0.55, 0.79	**<0**.**001**
Household income									
$20,000 to $39,999	0.69	0.49, 0.97	**0**.**031**	0.80	0.56, 1.13	0.197	0.79	0.56, 1.12	0.189
$40,000 to $59,999	0.52	0.37, 0.74	**<0**.**001**	0.57	0.40, 0.82	**0**.**002**	0.56	0.39, 0.81	**0**.**002**
$60,000 to $79,999	0.37	0.26, 0.53	**<0**.**001**	0.46	0.32, 0.66	**<0**.**001**	0.34	0.23, 0.49	**<0**.**001**
$80,000 or more	0.39	0.27, 0.55	**<0**.**001**	0.52	0.36, 0.74	**<0**.**001**	0.25	0.18, 0.36	**<0**.**001**
Dental insurance									
Insured	0.29	0.25, 0.34	**<0**.**001**	0.38	0.33, 0.43	**<0**.**001**	0.36	0.32, 0.41	**<0**.**001**
Immigration status									
Immigrant	1.57	1.36, 1.82	**<0**.**001**	1.40	1.21, 1.61	**<0**.**001**	0.79	0.68, 0.92	**0**.**003**
Disability * Sex									
Some difficulty * Female	1.04	0.82, 1.32	0.752	0.98	0.76, 1.26	0.891	1.06	0.84, 1.33	0.638

Bold *p*-values indicate statistical significance at α < 0.05.CI, Confidence Interval; OR, Odds Ratio.

a Reference categories: no difficulty; less than $20,000 household income; no insurance; male sex; non-immigrant.

Predicted probabilities from the interaction models are presented in Table 5 and illustrated in Figure 2. For cost-related visit avoidance, functional difficulty was associated with a roughly 7 percentage point increase in predicted probability among men (no difficulty: 16.4%, 95% CI 14.3–18.8%; some difficulty: 23.4%, 95% CI 21.1–25.8%) and a comparable 8 percentage point increase among women (no difficulty: 16.6%, 95% CI 14.3–19.2%; some difficulty: 24.3%, 95% CI 21.9–26.9%). A near-identical pattern was observed for treatment avoidance: the functional difficulty gap was 6.6 percentage points in men (13.5% vs. 20.1%) and 6.4 percentage points in women (13.6% vs. 20.0%), with confidence intervals overlapping substantially across sexes.

**Table 5:** Predicted probabilities of cost-related dental care avoidance and emergency-only or no dental visiting among Canadian adults aged ≥65 years by disability status and sex, 2022 CCHS.

**Disability**	**Sex**	**Dental visit avoidance due to cost**	**Dental treatment avoidance due to cost**	**Emergency-only or no dental visits**
No difficulty	Male	16.4% (14.3–18.8)	13.5% (11.6–15.7)	23.9% (21.4–26.6)
Some difficulty	Male	23.4% (21.1–25.8)	20.1% (18.0–22.3)	32.9% (30.1–35.8)
No difficulty	Female	16.6% (14.3–19.2)	13.6% (11.7–15.8)	17.1% (14.7–19.8)
Some difficulty	Female	24.3% (21.9–26.9)	20.0% (18.1–22.0)	25.4% (22.9–28.0)

Predicted probabilities and 95% confidence intervals (CI) were estimated from survey-weighted logistic regression models using marginal means.

The emergency-only or no dental visiting outcome revealed a distinct pattern. While the functional difficulty effect was again consistent across sexes (men: 23.9% without any functional difficulty vs. 32.9% with difficulty; women: 17.1% vs. 25.4%), women had substantially lower predicted probabilities of emergency-only or no dental visiting than men within each disability stratum. Among those without any functional difficulty, the predicted probability of emergency-only or no dental visiting was 23.9% in men vs. 17.1% in women, a 6.8 percentage point sex difference. Among those with functional difficulties, this gap was maintained: 32.9% in men vs. 25.4% in women, a difference of 7.5 percentage points. The disability increment and the sex difference thus operated as additive, independent effects, consistent with the non-significant interaction term. In absolute terms, these estimates indicate that older adults with functional difficulties were approximately 7–8 percentage points more likely to report cost-related avoidance of dental visits or treatment than those without functional difficulty, a difference that is meaningful at the population level in a nationally representative sample of older Canadians. Interaction plots (Figure 2) visually confirm the parallel trajectories across disability strata for both sexes across all three outcomes.

## 4 Discussion

The central question this paper explored was whether sex modifies the relationship between any functional difficulty and barriers to dental care among older Canadians. Our findings suggest no significant association for cost-related barriers, but sex matters independently for emergency-only or no dental visiting. Functional difficulty was associated with worse access across all three outcomes, and this disadvantage was similarly sized for both male and female groups. Women, however, were less likely to rely on emergency-only or no dental care regardless of functional difficulty status. What emerges is a picture in which functional difficulty operates as a broad structural constraint on dental care access, while sex shapes patterns of care-seeking within that broader picture.

### 4.1 Functional difficulty is associated with worse access in both sexes

The functional difficulty effect was consistent and sizeable across all three outcomes, and was meaningful in both absolute and relative terms. In the fully adjusted models, the odds of cost-related visit and treatment avoidance were about 58%–59% higher among those reporting functional difficulty, and the predicted probabilities suggest an absolute disadvantage of roughly 7–8 percentage points for both outcomes. In a population-level context, differences of this size imply that a substantial number of older adults with functional difficulty may be deferring needed dental care despite adjustment for income and insurance. What makes the functional difficulty effect particularly striking is that it did not disappear even after accounting for income and dental insurance. Insurance was the most powerful protective factor in the models; people with coverage were about 71% less likely to avoid a dental visit due to cost, yet functional difficulty remained a significant predictor even among the insured. That points toward something beyond finances: the practical difficulty of getting to a dental office, managing an appointment, or communicating with a provider when you are living with functional limitations (4, 5). While CDCP's focus on addressing the cost-related issues is a critical step forward, coverage alone might not narrow the residual functional difficulty gap if driven by non-financial barriers. This may warrant close attention in future implementation research.

The sequential sensitivity analyses in Supplementary Table S1 tell a consistent story: the functional difficulty effect attenuated modestly as income and education were added to the model, suggesting that socioeconomic position accounts for some of the gap, but the association was not reducible to socioeconomic position. The magnitude of these associations is consistent with broader literature showing that structural disadvantages such as low income, lack of insurance, and disability are associated with substantial barriers to dental care use rather than only marginal differences. Previous work on financial barriers and dental service inequalities has similarly shown that socially disadvantaged groups experience materially lower access to routine care and greater reliance on delayed or problem-oriented use (1, 2, 14). This interpretation also aligns with disability-focused literature showing that access barriers operate at multiple levels beyond the financial (4, 15).

### 4.2 Non-significant Sex-based effect modification for cost avoidance

The interaction tests came back non-significant across all three outcomes (visit avoidance: interaction OR 1.04, *p* = 0.752; treatment avoidance: OR 0.98, *p* = 0.891; emergency or no dental visiting: OR 1.06, *p* = 0.638), and the predicted probabilities make this concrete. The functional difficulty gap in cost-related visit avoidance was 7.0 percentage points for men (16.4% without any functional difficulty vs. 23.4% with) and 7.7 percentage points for women (16.6% vs. 24.3%). Those are essentially the same gap. The association between functional difficulty and cost-related dental care avoidance did not differ significantly by sex.

A null interaction warrants examination beyond its statistical insignificance. It suggests that regardless of the specific challenges facing those who identify with a functional difficulty, such as financial strain, logistical difficulties, or the navigation of services, the burden bears down with a similar weight on both men and women (16). If this interpretation holds up in future research, it might have practical implications regarding the program features designed to make dental care more accessible for people with functional difficulty regardless of the individual's sex. Both groups appear to face the same underlying structural constraints. That said, these findings ought to be examined within the context of the cross-sectional nature of this survey data.

The broader literature on sex and oral health has documented differences in oral health outcomes and care-seeking behaviours (2, 14), but fewer studies have examined the more specific question of whether sex changes how structural disadvantage, like functional difficulty, translates into access barriers. The present finding adds something to that conversation: it suggests that sex shapes individual patterns of dental care use (a point the next section addresses) but does not appear to change the size of the barrier that functional difficulty creates.

### 4.3 Women show lower odds of emergency-only or no dental visiting

Sex did not modify the functional difficulty penalty, but it did matter independently for one outcome: emergency-only or no dental visiting. Women were about 32% less likely than men to report emergency-only or no dental visiting in the fully adjusted model (OR 0.68, 95% CI 0.61–0.76, *p* < 0.001), and this difference was present within both functional difficulty strata. Among those without functional difficulties, 23.9% of men had emergency-only or no dental visiting patterns, compared to 17.1% of women. For those with functional difficulties, the rates rose to 32.9% for men and 25.4% for women. While functional difficulty increases the rate for both groups, men consistently reported higher emergency-only or no dental visits.

This is consistent with a pattern seen across health services research: women tend to engage with preventive and routine care more regularly than men across the life course (7, 8). The reasons are debated, including health literacy, social norms, and the role of reproductive health experiences in establishing early care habits (17, 18). It is worth noting that the “emergency-only or no dental visiting” variable may not be interpreted similarly among all respondents. For some this might mean genuine unmet need; others may simply not prioritize routine care. This heterogeneity warrants a cautious interpretation of the findings of this analysis.

For the CDCP, the practical implication is that older men, particularly those with functional difficulty, are the group most likely to be entering the program with emergency-only care patterns that carry high costs, complex treatment needs, and missed preventive opportunities (19). A coverage program that includes preventive services creates an opening to change that pattern, but only if it reaches this group. Outreach that works well for people already inclined toward routine dental care may not be enough.

The baseline sex difference in emergency-only or no dental visiting rates documented here has implications for how the CDCP's impact might be evaluated. If baseline rates of emergency visits differ between male and female groups, as these cross-sectional data suggest, then post-implementation comparisons would ideally be stratified by this variable to avoid masking differential patterns of program uptake or changes in care-seeking. A program that reaches those currently relying on emergency care as their only point of dental contact may produce different population-level effects than one that shifts already-preventive patterns, and aggregate statistics could obscure these distinctions. Sex-stratified surveillance using national survey data would support a more nuanced evaluation.

### 4.4 Immigration status as a compounding access barrier

The immigration finding deserves attention on its own terms. After adjusting for income and insurance, immigrant older adults had about 58% higher odds of avoiding a dental visit due to cost and 39% higher odds of avoiding recommended treatment, effects nearly as large as the functional difficulty effect itself. However, immigration was also significantly associated with emergency-only or no dental visiting in the opposite direction: immigrant older adults had 21% lower odds of reporting emergency-only or no dental visiting compared with non-immigrants (OR 0.79, 95% CI 0.68–0.92, *p* = 0.003). One possible interpretation is that immigrant older adults may delay or avoid certain forms of dental care because of financial, linguistic, cultural, or system-navigation barriers, while still maintaining some level of episodic or problem-oriented engagement with dental services (20).

The CDCP requires people to actively enroll through an online or telephone process, find a participating dentist, and navigate ongoing eligibility requirements. For someone unfamiliar with Canadian systems, facing language barriers, or without a regular care provider, those steps are not trivial. The elevated cost-avoidance rates among immigrant older adults suggest that this group was already being systematically under-served before the CDCP launched, and without deliberate outreach, that pattern could easily persist under the new program.

### 4.5 Strengths and limitations

A few features of this study are worth noting as strengths. The sample is large (*n* = 23,967) and nationally representative, with complex survey weights properly applied throughout. We formally tested the functional difficulty × Sex interaction rather than simply comparing subgroups, which is the appropriate method for the research question. Predicted probabilities are reported alongside odds ratios, which makes the results more interpretable. And the timing of the data, the 2022 CCHS cycle, collected immediately before the CDCP rollout, gives the findings direct policy relevance as a pre-implementation baseline. The sample size provided adequate power to detect the main effects of functional difficulty and to evaluate the precision of the null interaction estimates.

Several limitations are notable. The cross-sectional design prevents causal inference. Due to data limitations, gender identity could not be examined, and no distinction between sex and gender is possible within the available dataset. Accordingly, the analysis focuses on sex-based differences, while acknowledging that gender, as a broader social construct, may influence dental care access through pathways not captured in this study. Gender, as a multidimensional social construct encompassing norms, roles, identities, and relational dynamics, shapes dental care access through mechanisms not fully captured by a binary sex variable (21). Future studies using surveys that measure gender identity alongside sex are needed to characterize these relationships more completely. The exclusion of institutionalized individuals is a particularly consequential limitation for a study of disability and dental care access. Based on global studies, people living in long-term care facilities, hospitals, and other institutional settings may have substantially higher rates of both functional difficulty and unmet dental need than the community-dwelling population captured by the CCHS (22, 23). By excluding this group, the study effectively samples a healthier and more functionally independent subset of older adults with functional difficulties, producing a healthy-survivor bias that is likely to underestimate the true burden of disability-related dental inequity in Canada. The findings reported here should therefore be interpreted as conservative lower-bound estimates of the disability access gap, and future research using administrative or facility-level data will be needed to characterize the full population. The disability exposure was operationalized as a binary indicator of functional difficulty using the Washington Group Short Set, with “some difficulty” in at least one domain as the threshold. This produced a large exposed group (63% of the sample), reflecting the high prevalence of functional limitation across the full spectrum from mild to severe among adults aged 65 and older. Although consistent with population-based surveillance practice, this operationalization does not distinguish between mild, moderate, and severe functional limitations. A sensitivity analysis using a stricter threshold, for example, requiring “a lot of difficulty” or “cannot do at all” in at least one domain, was not feasible with the available PUMF data due to sample size constraints, but would be a valuable direction for future work using master file data. Self-reported dental care avoidance may be subject to recall and social desirability bias.

## 5 Conclusion

This study provides pre-CDCP baseline evidence that functional difficulty is a strong and consistent correlate of dental care access inequity among older Canadians. The magnitude of this disadvantage was similar across binary male/female groups, suggesting that functional difficulty-related barriers are broadly shared rather than strongly sex-differentiated in this population. At the same time, women had lower odds of emergency-only or no dental visiting, indicating that sex-related differences in dental care-seeking patterns may still matter for monitoring and evaluation.

These findings suggest that equitable CDCP implementation will require attention not only to financial coverage but also to whether older adults with functional difficulties can access and use care in practice. Continued surveillance will be important to determine whether disability-related access gaps narrow over time, and whether program gains are distributed equitably across disability status and sex.

## Data Availability

Publicly available datasets were analyzed in this study. This data can be found here: The data for this study are available from Statistics Canada through the Canadian Community Health Survey (CCHS): Public Use Microdata File (PUMF). Direct link: https://www150.statcan.gc.ca/n1/en/catalogue/82M0013X. No repository accession number applies.
