# Supplementary material for: Disability-related barriers to dental care among older Canadian adults: the role of sex as an effect modifier
Source: Front Oral Health. 2026 Jun 16;7:1835484. doi: 10.3389/froh.2026.1835484 (PMC13314637; doi:10.3389/froh.2026.1835484)
Supplement: Supplementary file 1 [file Table1.pdf]

Supplementary Material

Table S1. Logistic regression: Association between disability status and cost-related dental care avoidance outcomes among older adults, Canadian Community Health Survey (CCHS) 2022. (three sequential models).

Model 1: Adjusted for sex.

	Dental visit avoidance due to cost			Dental treatment avoidance due to cost			Emergency-only or no dental visits		
**Predictor**[1]	**OR**	**95% CI**	**p-value**	**OR**	**95% CI**	**p-value**	**OR**	**95% CI**	**p-value**
**Disability**									
Some difficulty	1.63	1.45, 1.83	**<0.001**	1.64	1.45, 1.85	**<0.001**	1.69	1.52, 1.87	**<0.001**
**Sex**									
Female	1.10	0.99, 1.23	0.082	1.05	0.93, 1.18	0.444	0.78	0.71, 0.86	**<0.001**

Abbreviations: CI = Confidence Interval, OR = Odds Ratio

[1]Reference categories: no difficulty; male sex.

Model 2: Adjusted for sex, household income, and education.

Predictor[1]	Dental visit avoidance due to cost			Dental treatment avoidance due to cost			Emergency-only or no dental visits		
	**OR**	**95% CI**	**p-value**	**OR**	**95% CI**	**p-value**	**OR**	**95% CI**	**p-value**
**Disability**									
Some difficulty	1.53	1.36, 1.73	**<0.001**	1.56	1.38, 1.77	**<0.001**	1.54	1.38, 1.71	**<0.001**
**Sex**									
Female	1.05	0.94, 1.17	0.412	1.01	0.90, 1.14	0.852	0.68	0.61, 0.76	**<0.001**
**Household income**									
$20,000 to $39,999	0.68	0.49, 0.95	**0.022**	0.80	0.56, 1.14	0.220	0.75	0.54, 1.03	0.079
$40,000 to $59,999	0.48	0.34, 0.67	**<0.001**	0.52	0.36, 0.74	**<0.001**	0.53	0.38, 0.75	**<0.001**
$60,000 to $79,999	0.31	0.22, 0.44	**<0.001**	0.37	0.26, 0.54	**<0.001**	0.33	0.23, 0.47	**<0.001**
$80,000 or more	0.30	0.21, 0.42	**<0.001**	0.39	0.27, 0.56	**<0.001**	0.24	0.17, 0.33	**<0.001**
**Education**									
Post-secondary	0.95	0.84, 1.08	0.439	1.16	1.02, 1.33	**0.027**	0.52	0.46, 0.58	**<0.001**

Abbreviations: CI = Confidence Interval, OR = Odds Ratio

[1]Reference categories: no difficulty; male sex; less than $20,000 income; less than post-secondary education.

Model 3: Adjusted for sex, household income, education, immigration status, and dental insurance.

Predictor[1]	Dental visit avoidance due to cost			Dental treatment avoidance due to cost			Emergency-only or no dental visits		
	**OR**	**95% CI**	**p-value**	**OR**	**95% CI**	**p-value**	**OR**	**95% CI**	**p-value**
**Disability**									
Some difficulty	1.58	1.39, 1.78	**<0.001**	1.59	1.40, 1.80	**<0.001**	1.58	1.41, 1.77	**<0.001**
**Sex**									
Female	1.05	0.94, 1.18	0.394	1.01	0.90, 1.14	0.835	0.67	0.60, 0.75	**<0.001**
**Household income**									
$20,000 to $39,999	0.67	0.47, 0.94	**0.022**	0.80	0.56, 1.14	0.209	0.73	0.51, 1.04	0.081
$40,000 to $59,999	0.51	0.35, 0.72	**<0.001**	0.55	0.38, 0.80	**0.002**	0.57	0.39, 0.83	**0.003**
$60,000 to $79,999	0.37	0.26, 0.53	**<0.001**	0.44	0.30, 0.63	**<0.001**	0.38	0.26, 0.55	**<0.001**
$80,000 or more	0.38	0.26, 0.55	**<0.001**	0.48	0.33, 0.70	**<0.001**	0.30	0.21, 0.43	**<0.001**
**Education**									
Post-secondary	0.97	0.85, 1.10	0.590	1.19	1.03, 1.36	**0.014**	0.54	0.48, 0.60	**<0.001**
**Dental insurance**									
Insured	0.29	0.25, 0.34	**<0.001**	0.38	0.33, 0.43	**<0.001**	0.37	0.32, 0.42	**<0.001**
**Immigration status**									
Immigrant	1.61	1.39, 1.86	**<0.001**	1.39	1.21, 1.61	**<0.001**	0.84	0.72, 0.98	**0.030**

Abbreviations: CI = Confidence Interval, OR = Odds Ratio

[1]Reference categories: no difficulty; male sex; less than $20,000 income; less than post-secondary education; no dental insurance; non-immigrant.
